# Evaluation of antioxidative and diabetes-preventive properties of an ancient grain, KAMUT^®^ khorasan wheat, in healthy volunteers

**DOI:** 10.1007/s00394-017-1579-8

**Published:** 2017-11-16

**Authors:** Caterina Trozzi, Francesca Raffaelli, Arianna Vignini, Laura Nanetti, Rosaria Gesuita, Laura Mazzanti

**Affiliations:** 1 Bioaesis srl, Jesi, AN Italy; 2 Biomedfood srl, Spinoff Università Politecnica delle Marche, Via Ranieri n.65, 60128 Ancona, Italy; 3 Dipartimento di Scienze Cliniche Specialistiche ed Odontostomatologiche, Università Politecnica delle Marche, Ancona, Italy; 4 Centro Interdipartimentale di Epidemiologia, Biostatistica e Informatica medica, Università Politecnica delle Marche, Ancona, Italy

**Keywords:** KAMUT^®^ khorasan wheat, Antioxidant properties, Selenium, DHA, Diabetes risk factors, Ancient wheat.

## Abstract

**Purpose:**

Recently, there was an increasing interest on the use of ancient grains because of their better health-related composition. The aim of this study was to evaluate in healthy human subjects the antioxidative and diabetes-preventive properties of ancient KAMUT^®^ khorasan wheat compared to modern wheat.

**Methods:**

The study was a randomized, non-blind, parallel arm study where the biochemical parameters of volunteers with a diet based on organic whole grain KAMUT^®^ khorasan products, as the only source of cereal products were compared to a similar replacement diet based on organic whole grain modern durum wheat products. A total of 30 healthy volunteers were recruited and the intervention period lasted 16 weeks. Blood analyses were performed before and after the diet intervention. The effect of KAMUT^®^ khorasan products on biochemical parameters was analyzed by multiple quantile regression adjusted for age, sex, physical activity and BMI compared to data at baseline.

**Results:**

Subjects receiving KAMUT^®^ khorasan products showed a significantly greater decrease of fat mass (*b* = 3.7%; CI 1.6–5.5; *p* = 0.042), insulin (*b* = 2.4 µU/ml; CI 0.2–4.2; *p* = 0.036) and a significant increase of DHA (*b* = − 0.52%; CI − 1.1 to − 0.12; *p* = 0.021).

**Conclusions:**

Our study provides evidence that a substitution diet with KAMUT^®^ khorasan wheat products can reduce some markers associated to the development of type-2 diabetes compared to a diet of modern wheat.

## Introduction

Recent studies have demonstrated an increase in consuming whole grain and its derivative products associated with a reduced risk to develop chronic diseases, i.e. cancer, cardiovascular disease, diabetes and obesity [1, 2]. Different mechanisms are possible for this protection because whole cereals are rich in nutrients and phytochemicals. In particular, whole cereals are rich in antioxidants, including phenolic compounds, and in minerals [3]. In whole grain there are several types of antioxidant compounds with different structures and types of action. They assist in antioxidants indirectly by way of cofactors such as Iron, Zinc, Copper and Selenium, and directly as antioxidants by way of antioxidant compounds, such as ferulic acid, other polyphenols, carotenoids and vitamin E. It is assumed that they act in a synergistic way and that the antioxidant properties of each whole grain-based product rely on its peculiar antioxidant profile [4].

Growing interest in ancient grains appears in recent literature, because of their nutritional and health properties, and in particularly on KAMUT^®^ khorasan wheat. KAMUT^®^ is a registered trademark used to market an ancient variety of wheat called khorasan wheat (*Triticum turgidum* spp. *turanicum*). Khorasan wheat is genetically similar to modern durum wheat with origins that are claimed to be much older. Any khorasan wheat sold under the KAMUT^®^ brand must follow several quality specifications related both to nutritional characteristics and growing conditions (i.e. the grain must be grown organically) [5].

Some studies demonstrated that KAMUT^®^ khorasan wheat has a high carotenoids content [6] and a unique nutraceutical value for its peculiar content in bioactive phytochemicals [7]. Furthermore, KAMUT^®^ khorasan wheat is rich in selenium. It has been demonstrated that selenium content in KAMUT^®^ khorasan bread was ten-fold higher than modern durum bread [8, 9]. Selenium acts in the active site of several enzymes involved in cellular protection from oxidative damage, such as glutathione peroxidase and other selenoproteins [10].

In two previous studies [4, 8], after 7 weeks of experimental diets which were based on either ancient or modern wheat bread, with or without sourdough fermentation, rats from these groups were further divided into 2 subgroups, one receiving an oxidative stress agent by doxorubicin injection. The highest protective effect against oxidative stress was seen in KAMUT^®^ khorasan bread fed animals, compared to modern wheat bread fed animals, especially when a KAMUT^®^ sourdough bread was supplied. Sourdough fermentation could have increased the levels of easy-extractable phenolic compounds as well as the vitamin E and beta-carotene contents and these modifications could have significantly contributed to the observed protective activity of KAMUT^®^ sourdough bread.

From these results, they supposed that KAMUT^®^ khorasan wheat is useful to increase antioxidant protection. Moreover, histological evaluation of the hepatic tissue evidenced the onset of inflammation in response to doxorubicin only in modern wheat fed rats. This potential anti-inflammatory properties of KAMUT^®^ khorasan wheat has been further confirmed in another trial with pasta fed rats [11]. In both basal and after an oxidative stress by doxorubicin injection, the histological evaluation of the duodenum morphology of modern wheat pasta fed rats evidenced a flattened mucosa, an unusual shape and shortening of the villi, and a high lymphocyte infiltration, while no modifications were detected in KAMUT^®^ khorasan pasta fed animals. The antioxidative and anti-inflammatory properties of KAMUT^®^ khorasan wheat compared to modern wheat were further confirmed in cultured liver cells supplemented with in vitro digested cookies [12].

Two clinical trials evaluated the impact of diet on cardiovascular risk factors in both healthy subjects [13] and in patients suffering from Acute Coronary Syndrome [14] and evidenced improved metabolic, antioxidant and inflammatory blood profiles after a KAMUT^®^ khorasan wheat-based diet compared to a modern wheat-based diet. Another human clinical study on patients suffering from Irritable Bowel Syndrome (IBS) [15] evidenced that consumption of KAMUT^®^ khorasan products decreased the extent and severity of symptoms related to IBS and their blood inflammatory profile. A recent study showed that a KAMUT^®^ khorasan wheat-based diet, compared to a modern wheat-based diet, was effective in the secondary prevention care in 21 type-2 diabetes mellitus patients by reducing total and LDL cholesterol, insulin, blood glucose, ROS production and some inflammatory risk factors [16].

Except for the studies just cited above, almost all previous studies regarding antioxidative and diabetes-preventive properties of cereals were in vitro based on raw materials such as flour, wheat germ, bran, instead of finished products.

The aim of the present study was to evaluate in vivo, in healthy human subjects, the antioxidative and diabetes-preventive properties of food made of whole grain, and to compare differences between ancient and modern wheat.

KAMUT^®^ is a registered trademark of Kamut International, Ltd. and Kamut Enterprises of Europe, bvba.

## Materials and methods

### Selection of volunteers

Healthy volunteers were recruited from Jesi (Ancona, Italy) and nearby communities by BIOAESIS S.r.l. Laboratory, via newspaper advertisements and direct mailings. Subjects were selected according to the following inclusion criteria: age ranging between 25 and 60 years, good general health, not pregnant nor lactating, no irregular menses, nor fertility problems, a varied diet not based on KAMUT^®^ khorasan products and alcoholic consumption of one-half litre per day at most, no gluten allergy or wheat gluten severe sensitivity, nor food disorders, no smokers, no athletes, no shift workers and no prescription of drugs that could interfere with the project (for example drugs for hypertension).

Forty five potential participants meeting these criteria were analyzed for protein C-reactive, omega3/omega6 fatty acids (AA/EPA, AA/DHA), D-ROMs test (Reactive Oxygen Metabolites test), BAP test (Biological Antioxidant Potential test) and according to these results 30 subjects who had no more than one of these parameters values outside but close to the normal ranges were finally included in the project. Normal ranges were considered to be: protein C-reactive < 0.100 mg/dl, AA/EPA 1.00–3.00; AA/DHA 0.50–2.00; D-ROMs < 300 U.carr and BAP test < 2200 µmol/l.

The low number of volunteers selected was due to the fact that this study was considered to be a pilot study.

### Study design and intervention

The study was a randomized, non-blind, parallel arm study where the biochemical parameters of a group of volunteers with a diet based on KAMUT^®^ khorasan products as the only source of cereal (KAMUT^®^ group) was compared to a similar replacement diet in a group of volunteers with a diet based on modern durum wheat products as their only source of cereal (Control group).

At the beginning of the intervention study, the 30 recruited volunteers (26 females and 4 males) were randomly divided into the two groups, KAMUT^®^ and Control group, 15 subjects in each group. Regarding the 4 males, half of them (2 males) were assigned to one group and the other 2 males to the other group. The males were randomly assigned to each group. Five (33.3%) and seven (46.7%) subjects, respectively, in KAMUT^®^ and Control groups, did regular physical activity.

The intervention period lasted 16 weeks for both groups. Each group was allowed to eat cereal products other than the ones provided by the study no more than one day per week. During the other 6 days the only cereal source had to be the products provided by the study. These products included bread, biscuits, two types of pasta (fusilli and penne), crackers and crisp toasts. Participants were advised to keep the cereal products daily intake constant during the test period, including those days when they ate cereal products other than the ones provided by the study. No other modifications regarding their normal diet before recruitment were done. Moreover, the subjects were advised to maintain their normal body weight and other living habits during the intervention period. All participants received written and oral instructions concerning the diets before the test period.

The male participants in each group received 350 g/week of fusilli, 350 g/week of penne, 420 g/week of bread, 300 g/week of biscuits, 180 g/week of crisp toasts, 100 g/week of crackers. The female participants in each group received 250 g/week of fusilli, 250 g/week of penne, 300 g/week of bread, 150 g/week of biscuits, 100 g/week of crisp toasts, 100 g/week of crackers.

Diets were proportionally equivalent in macro and micronutrient quantity for all the subjects, containing 100% of the recommended daily allowance (RDA) for all nutrients.

Written informed consent was obtained from each participant before the beginning of the study. The study protocol was approved by Università Politecnica delle Marche (Italy) Ethics Committee (Protocol No. 212267) and was conducted in accordance with the principles of the Declaration of Helsinki as revised in 2001.

### Wheat varieties

KAMUT^®^ group consumed products based on the ancient KAMUT^®^ khorasan wheat organically grown in Montana (USA) and in Saskatchewan (Canada). Control group consumed products based on a mix of modern commercial Italian durum wheat varieties organically grown in Italy. Both type of grain were milled by BAREA S.R.L. (Quinto di Treviso, Treviso, Italy) and one lot of each kind of flour was produced and purchased. The two flours obtained were used by the bakery Fiordelmondo (Jesi, Ancona, Italy) to prepare weekly bread and biscuits utilizing a sourdough process.

Crackers, pasta and crisp toasts were prepared by PROBIOS s.r.l. (Campi Bisenzio, Firenze, Italy) and each kind of product was from the same lot of production.

All the food production procedures were identical for both KAMUT^®^ khorasan and control products to further standardize the comparison.

### Food analysis

All the KAMUT^®^ khorasan and control products (fusilli, penne, crackers, crisp toast, flour) were analyzed for primary component composition, for various secondary metabolites with antioxidant properties and for Selenium. Fatty acids were determined in flours samples only.

The energy value was estimated by calculation according to the Commission Directive 2008/100/EC. Carbohydrates were determined by calculation. The carotene content (13-cis-beta-Carotene, all-trans-alpha-Carotene, all-trans-beta-Caroten, 9-cis-beta-Carotene) was estimated from the yellow pigment content, extracted and the pigments were measured by RP-HPLC. The total folic acid content was estimated using a microbiological turbidimetric assay using the Enterococcus hirae ATCC 8043 strain. The vitamin E content was analyzed on the extracts by HPLC. The soluble, insoluble and total dietary fibre content was measured according to the AOAC 991.43 1994 method. Selenium (Se) was extracted by nitric acid digestion and the analyses were carried out by ICP-MS. The total polyphenol content was measured at 750 nm using the spectrophotometric Folin–Ciocalteu method with gallic acid as the reference standard. The moisture content was measured according to the CEE REG 824/2000 19/04/2000 All IV method for the flour, the ISO 712:2009 method for pasta products and the ISTISAN REPORT 1996/34 Page 7 method B for the other products. The protein content was measured according to the AOAC 992.23 1992 method. The total fat content was measured according to the DM 23/07/1994 SO GU N°186 10/08/1994 method for the pasta products and the ISTISAN REPORT 1996/34 page 41 method A for the other products. The ash content was measured according to the AOAC 923.03 1923 method for the flour and the ISTISAN REPORT 1996/34 page 77 method for the other products. The fatty acids AA, EPA, DHA and ALA were measured according to the ISTISAN REPORT 1996/34 page 47 method.

### Blood analysis

Before and after diet intervention, venous blood samples were drawn, after an overnight fasting, from all volunteers enrolled in the study at Bioaesis srl laboratory (Jesi, Ancona, Italy). The day before, subjects were asked to strictly follow the diet and to avoid heavy exercise and unusual large portions of food. The subjects were asked to arrive to the laboratory by car or by bus to avoid extra physical stress.

Serum samples were collected into evacuated plastic tubes CLOT ACTIVATOR VACUTEST PLAST and plasma samples were collected in tubes containing K_3_ EDTA 5.4 mg VACUTEST PLAST (VACUTEST KIMA srl, Arzergrande, Padova, Italy) for the measurement of fatty acids and plasmatic homocysteine. Blood samples obtained from each subject were centrifuged at 1400 g for 10 min to separate serum or plasma and then processed.

Lipid variables, blood glucose, serum electrolytes were determined by conventional methods.

Antioxidant barrier was assessed by performing a photometric test, the Biological Antioxidant Potential (BAP test, Diacron srl, Grosseto, Italy).

Plasma levels of Reactive Oxygen Metabolites (ROM) were measured by utilizing a photometric test, the dROMs Test kit (Diacron srl, Grosseto, Italy).

Serum levels of insulin were quantified using the Chemiluminescence Microparticle Immuno Assay (CMIA) ARCHITECT insulin (ABBOTT, Wiesbaden, Germany) following the manufacturer’s protocol.

BAP test, dROMs test and serum levels of insulin were quantified using the ARCHITECT CI 8200 instrument (ABBOTT, Wiesbaden, Germany).

The Fatty acids AA, EPA, DHA were determined according to Marangoni [17] and Risé [18].

### Fecal and urine samples collection

Each participant was asked to collect a fecal and urine sample before entering the study and after 16 week dietary intervention. Samples were immediately frozen at − 80 °C. Phylogenetic microarray platform High Taxonomic Fingerprint (HTF)—Microbi.Array analysis was used to explore the fecal microbiota and solid-phase microextraction-gas chromatography-mass spectrometry (SPME-GC-MS) analysis was used to explore the metabolic profiles on both fecal and urine samples as described by Taneyo Saa [19].

### Compliance and health status monitoring

At the beginning of the study, patients underwent a medical examination and an interview to obtain information about personal medical history, demographics, medication, and lifestyle habits. This descriptive supplementary information was not used as a basis for patient exclusion, but rather was used for monitoring the health status of each volunteer during the diet intervention.

Body composition was measured at the beginning and at the end of the project during the medical examination using bioelectrical impedance analysis (BIA) (Human Im Plus II, DS Medica, Milano, Italy) following the manufacturer’s instruction. Before the test period all participants received specific instructions about how to prepare before BIA analysis: (1) not to eat or drink within 4 h before the test, (2) to maintain normal body hydration, (3) not to consume caffeine or alcohol within 12 h before the test, (4) not to exercise within 6 h before the test, (5) not to take diuretics within 7 days before the test, (6) to urinate within 30 min before the test. The adherence to these instructions were assessed with a questionnaire by the physician before starting the BIA test.

All the volunteers were strictly monitored and the compliance with the study and their general health status were assessed weekly by individual interviews and monthly by medical examinations. Moreover, they received additional advice concerning the diets during the weekly individual interviews and the monthly medical examinations. The compliance with the study in terms of maintaining the normal body weight and their general health status was assessed monthly by medical examination where body weight, blood pressure, and BMI were also measured. Moreover, during the 16 weeks of experimental diet volunteers wrote a diary where they noted all the food they had eaten. An additional form was filled out weekly describing symptoms of possible concurrent illnesses or anything else unusual regarding subject’s experience in consuming the experimental food products. The compliance with the study in terms of maintaining the living habits and the adherence to the diets was assessed weekly during the individual interviews via self-report food diaries and additional forms, as described above.

### Statistical analysis

A non-parametric approach was followed due to the small group size. Comparisons between groups at the baseline were performed by means of Wilcoxon rank sum test and variables were summarized using percentiles and graphically represented in boxplots.

The effect of KAMUT^®^ khorasan products on biochemical parameters was analyzed by multiple quantile regression. The absolute change between T0 and T4 in each biochemical variable was the dependent variable, diet based on KAMUT^®^ khorasan or durum modern wheat was the independent variable of interest and age, sex, physical activity and BMI at baseline were the covariates. Results were expressed as estimated regression coefficients and 95% Confidence Intervals (CI). All the analyses were performed using the R statistical package and a probability of 5% was used to assess the statistical significance.

## Results

### Food analysis

KAMUT^®^ khorasan and control products were analysed for primary component composition, for various secondary metabolites with antioxidant properties and for selenium and the results are listed in Table 1. Fatty acids were determined in flour samples only and the results are found in Table 2.

**Table 1:** Food analysis

	KAMUT^®^ khorasan *(crisp toast)*	Control *(crisp toast)*	*p*	KAMUT^®^ khorasan *(crackers)*	Control *(crackers)*	*p*	KAMUT^®^ khorasan *(fusilli)*	Control *(fusilli)*	*p*	KAMUT^®^ khorasan *(penne)*	Control *(penne)*	*p*	KAMUT^®^ khorasan *(flour)*	Control *(flour)*	*p*
Humidity, g/100 g	3.10 (0.19)	4.93 (0.19)	< 0.001	2.87 (0.19)	1.45 (0.19)	< 0.001	9.96 (0.15)	10.28 (0.15)	< 0.001	9.68 (0.15)	10.96 (0.15)	< 0.001	10.58 (0.15)	10.59 (0.15)	0.883
Protein (N × 6.25), g/100 g	14.20 (0.50)	14.80 (0.55)	0.020	13.58 (0.49)	10.84 (0.39)	< 0.001	14.30 (0.50)	11.53 (0.42)	< 0.001	14.00 (0.50)	11.57 (0.42)	< 0.001	13.36 (0.48)	10.40 (0.38)	< 0.001
Fat, g/100 g	7.32 (0.39)	5.96 (0.32)	< 0.001	10.99 (0.58)	10.88 (0.58)	0.677	1.66 (0.08)	1.96 (0.09)	< 0.001	1.59 (0.07)	1.91 (0.09)	< 0.001	1.72 (0.09)	2.07 (0.11)	< 0.001
Ashes g/100 g	3.17 (0.08)	2.55 (0.07)	< 0.001	3.07 (0.08)	3.25 (0.09)	< 0.001	1.60 (0.04)	1.60 (0.04)	1.000	1.56 (0.04)	1.49 (0.04)	0.001	1.84 (0.02)	1.79 (0.02)	< 0.001
Carbohydrates, g/100 g	62.31 (0.87)	61.46 (0.87)	0.042	64.59 (0.84)	67.88 (0.81)	< 0.001	63.68 (0.73)	65.73 (0.68)	< 0.001	65.07 (0.70)	66.07 (0.64)	0.004	62.30 (0.75)	64.65 (0.73)	< 0.001
Energetic value, kcal/100 g	392 (3)	379 (2)	< 0.001	421 (3)	424 (3)	0.038	344 (2)	344 (2)	1.000	347 (2)	344 (2)	0.004	339 (2)	340 (2)	0.278
Total dietary fiber, g/100 g	9.9 (0.6)	10.3 (0.6)	0.153	4.9 (0.3)	5.7 (0.4)	< 0.001	8.8 (0.5)	8.9 (0.5)	0.660	8.1 (0.5)	8.0 (0.5)	0.660	10.2 (0.6)	10.5 (0.6)	0.278
Insoluble dietary fiber, g/100 g	7.7 (0.5)	7.0 (0.4)	0.003	4.1 (0.3)	4.7 (0.3)	< 0.001	6.1 (0.4)	6.0 (0.4)	0.583	5.3 (0.4)	5.6 (0.4)	0.111	7.2 (0.4)	7.5 (0.5)	0.156
Soluble dietary fiber, g/100 g	2.2 (0.2)	3.3 (0.3)	< 0.001	0.8 (0.2)	1.0 (0.2)	0.038	2.7 (0.3)	2.9 (0.3)	0.153	2.8 (0.3)	2.4 (0.2)	0.003	3.0 (0.3)	3.0 (0.3)	1.000
Folic acid, μg/100 g	36.0 (2.5)	40.0 (2.6)	0.003	29.0 (2.3)	15.0 (2.1)	< 0.001	35.0 (2.4)	37.0 (2.5)	0.085	31.0 (2.4)	27.0 (2.3)	0.001	31.0 (2.4)	25.0 (2.2)	< 0.001
Vitamin E, mg/100 g	24.60 (1.50)	25.80 (1.60)	0.101	4.50 (0.28)	5.09 (0.32)	< 0.001	0.12 (0.01)	0.29 (0.02)	< 0.001	0.20 (0.02)	0.21 (0.02)	0.278	1.03 (0.07)	0.49 (0.03)	< 0.001
Total polyphenols, mg/g DM	1.58 (0.08)	1.69 (0.08)	0.007	1.89 (0.09)	1.19 (0.06)	< 0.001	1.20 (0.06)	1.47 (0.07)	< 0.001	1.14 (0.06)	1.40 (0.07)	< 0.001	1.53 (0.08)	1.38 (0.07)	< 0.001
Selenium, mg/kg	1.12 (0.14)	0.10 (0.01)	< 0.001	1.02 (0.13)	0.11 (0.01)	< 0.001	1.28 (0.16)	0.13 (0.02)	< 0.001	1.23 (0.16)	0.10 (0.01)	< 0.001	1.09 (0.14)	0.09 (0.01)	< 0.001
13-cis-beta-Carotene, mg/100 g	< 0.01	< 0.01		< 0.01	< 0.01		< 0.01	< 0.01		< 0.01	< 0.01		< 0.01	< 0.01	
All-trans-alpha-Carotene, mg/100 g	< 0.01	< 0.01		< 0.01	< 0.01		< 0.01	< 0.01		< 0.01	< 0.01		< 0.01	< 0.01	
All-trans-beta-Carotene, mg/100 g	< 0.01	< 0.01		< 0.01	< 0.01		< 0.01	< 0.01		< 0.01	< 0.01		< 0.01	< 0.01	
9-cis-beta-Carotene, mg/100 g	< 0.01	< 0.01		< 0.01	< 0.01		< 0.01	< 0.01		< 0.01	< 0.01		< 0.01	< 0.01	

Data are reported as mean (SD); *p* values referred to *t* test for independent sample

**Table 2:** Fatty acid composition of KAMUT^®^ khorasan and control flour

	KAMUT^®^ khorasan (flour)	Control (flour)	*p*
Arachidonic acid (AA), g/100 g	< 0.2	< 0.2	
Eicosapentaenoic acid (EPA), g/100 g	< 0.2	< 0.2	
Docosahexaenoic acid (DHA), g/100 g	< 0.2	< 0.2	
Linolenic acid (ALA), g/100 g	16.2 (0.6)	44.1 (1.6)	< 0.001

Data are reported as mean (SD); *p* values referred to *t* test for independent sample

The energetic value appeared different among the various products. There was not any difference between the KAMUT^®^ khorasan fusilli and flour with respect to the corresponding control products. KAMUT^®^ khorasan crisp toast and penne showed a higher energetic value while KAMUT^®^ khorasan crackers showed a lower energetic value with respect to the corresponding control products. A significantly higher protein content was observed in all the KAMUT^®^ khorasan products with respect to the control products as previously reported [8, 13], except for the crisp toast. The ash content was higher in almost all the KAMUT^®^ khorasan products. Since all the food production procedures were identical and were carried out by the same manufacturer for both kinds of wheat products, this difference in ash content can be ascribed to a higher mineral content of KAMUT^®^ khorasan wheat compared to modern wheat, as confirmed in previous studies [13–16]. An averaged 10-fold higher value of selenium in all the KAMUT^®^ khorasan products was observed and this is similar to previous reported data [8, 12]. Selenium content in foods greatly depends on its concentration in the soil [20] and Se concentration in North American soil is reported to be higher than in Italy [21]. This can in part explain the higher Se content of KAMUT^®^ khorasan wheat which is always cultivated in North America compared to the Italian modern wheat varieties used in this study. Moreover, the concentration of the other metabolites with potential antioxidant activity appeared different among the various products. Total polyphenols were significantly higher in KAMUT^®^ khorasan flour and crackers while were significantly lower in KAMUT^®^ khorasan pasta and crisp toast. The content of Vitamin E was significantly higher in KAMUT^®^ khorasan flour and lower in KAMUT^®^ khorasan crackers and fusilli compared to control products. A significant difference was found in linolenic acid (ALA) content which was almost 3 times higher in the control flour than in KAMUT^®^ khorasan flour. No differences were observed in fiber content between KAMUT^®^ khorasan and modern wheat products.

### Characterization of the participants

The two groups of volunteers were comparable in age (Control: median age 37 yrs, 1st–3rd quartile 34–42 years; KAMUT^®^: median age 37 years, 1st–3rd quartile 36–44 years; *p* = 0.493) and BMI (Control: median BMI 22 kg/m^2^, 1st–3rd quartile 21–23 kg/m^2^; KAMUT^®^: median BMI 23 kg/m^2^, 1st–3rd quartile 22–25 kg/m^2^; *p* = 0.130) distribution. Subjects eating KAMUT^®^ khorasan products showed significant higher fat mass values (*p* = 0.019) and lower BAP test levels (*p* = 0.040) at baseline. No significant differences were found in the other biochemical parameters at baseline.

Blood pressure, body weight and BMI did not change significantly with respect to baseline in both groups at the end of diet (data not shown).

### Compliance and subjective experience

Compliance during the study was good: all the 30 subjects completed the study, wrote the diary recording all the food eaten, filled a form about their general health status and the experience of the food products consumed. The patients reported not having changed either medication or lifestyle habits (physical activity) or the amount of food intake consumed before the study during the course of the diet intervention.

During the diet, all the volunteers gave positive feed-back about their health status, reporting for example decreased hunger, a longer feeling of satiety, decreased constipation, decreased tiredness and decreased bloating. These observations were probably due to the increase of fiber intake in both groups independent of the habitual diet before the study began. However, the KAMUT^®^ group showed the best improvement and the best feeling with the food, assessed by a questionnaire provided in the additional form that volunteers filled weekly.

### Modification in biochemical parameters

The effect of KAMUT^®^ khorasan products on biochemical variables was analyzed by multiple quantile regression adjusted for age, sex, physical activity and BMI at baseline.

Table 3 shows the results of quantile regression analysis. Consumption of KAMUT^®^ products significantly affected the absolute changes in fat mass, DHA and insulin between baseline and after 16 weeks; subjects receiving KAMUT^®^ khorasan products showed a significantly greater decrease of fat mass (*b* = 3.7%; CI 1.6–5.5; *p* = 0.042) and insulin (*b* = 2.4 µU/ml; CI 0.2–4.2; *p* = 0.036) and a significant increase of DHA (b=-0.52%; CI -1.1 – -0.12; *p* = 0.021).

**Table 3:** KAMUT^®^ khorasan products consumption effects on absolute change of biochemical parameters

Dependent variables (absolute change)	Parameter estimate, *b*	95% CI
Fat mass, %	3.70	1.56; 5.46
BAP test, µmol Fe2+/l	− 102.28	− 241.02; 118.98
dROMs, U.carr	28.88	− 17.55; 60.09
Arachidonic acid (AA) %	− 0.53	− 1.05; 0.51
Eicosapentaenoic acid (EPA) %	0.13	− 0.34; 0.23
Docosahexaenoic acid (DHA) %	− 0.52	− 1.08; − 0.12
Total cholesterol, mg/dl	− 3.76	− 10.25; 25.84
LDL cholesterol, mg/dl	4.04	− 25.19; 14.06
HDL cholesterol, mg/dl	1.87	− 6.01; 4.25
Triglycerides, mg/dl	14.80	− 5.86; 24.17
Homocysteine, µmol/l	0.58	− 1.47; 1.28
Glycemia, mg/dl	0.19	− 1.94; 3.51
Insulin, µU/ml	2.38	0.19; 4.2
Metabolism, Kcal	− 6.32	− 62.5; 29.5
AST, U/I	− 0.40	− 3.8; 3.76
ALT, U/I	0.51	− 4.45; 2.53
Prot C, mg/dl	0.01	− 0.12; 0.05

Results of multiple quintile regression, adjusted for age, gender, physical activity and BMI

*BAP* biological antioxidant potential, *ROM* reactive oxygen metabolites, *LDL* low-density lipoprotein, *HDL* high-density lipoprotein, *AST* ASpartate aminoTransferase, *ALT* ALanine aminoTransferase, *Prot C* C-reactive protein

## Discussion

The Docosaesaenoic Acid (DHA) is a semi-essential omega-3 that is synthesized by the organism from Linolenic Acid (ALA) or can be introduced by the diet. Many experimental studies have shown the role played by DHA, together with other omega-3 (like EPA), in suppressing the development of most cancer processes [22–24] and in prevention and management of pathological conditions such as Alzheimer disease, coronary disease, hypertension, diabetes, arthritis and other inflammatory and autoimmune diseases [22, 23].

After 16 weeks of diet intervention the group eating KAMUT^®^ khorasan food showed a statistically significant increase in the concentration of serum DHA. Interestingly, in a clinical study enrolling 92 patients with non-alcoholic fatty liver disease and testing the effects of 15–18 months treatment with n-3 polyunsaturated fatty acids, the DHA percentage change (standardized *β*-coefficient − 0.19, *p* = 0.027) was independently associated with a decreased liver fat [25]. In another clinical study where the authors involved healthy people and patients with metabolic syndrome and coronary artery disease, they showed that AA and DHA were the main contributors to the cardiometabolic risk [adjusted *β*-coefficients (*β*′) for AA: 0.336, *p* < 0.001; for DHA: − 0.296, *p* < 0.001] and that DHA may be effective on suppression of vascular proliferation and inflammation [26].

In this study, a statistically significant increase in the concentration of serum DHA was shown in the KAMUT^®^ group compared to the control, but not in the concentration of serum EPA. The precursor of DHA is ALA and its concentration was higher in modern durum wheat flour than in KAMUT^®^ khorasan wheat flour, moreover DHA and EPA levels were undetectable in both KAMUT^®^ khorasan wheat and modern wheat flour (Table 2). Thus, the higher serum DHA’s concentration in KAMUT^®^ group cannot be explained with a higher introduction of its precursor or with a higher introduction of DHA and EPA with the experimental food. Moreover, all the volunteers were controlled for fatty fish consumption during the experimental diets. The synthesis of EPA from ALA involves the same first steps of the synthesis of DHA from ALA, but the synthesis of DHA is partly hormone-dependent contrary to the synthesis of EPA from ALA [27]. Since the KAMUT^®^ group did not show an increase in EPA level, it could not be excluded that the higher content of serum DHA is due to a higher presence in KAMUT^®^ khorasan products of compounds with estrogen-like activity such as polyphenols. Some papers reported that cereal flavonoids and wheat aleurone polyphenols increased plasma EPA and DHA concentrations in rats [28, 29]. In this work, only the total polyphenol content was measured so, it is not possible to draw final conclusions since specific classes of polyphenols with estrogen-like activity (lignans, for instance) were not evaluated. Selenium (Se) is an essential trace element with a high importance in humans’ health: Se is contained in catalytic centre of proteins [30], involved in immune function enhancement [31] and in cancer risk reduction [32, 33]. A recent study has demonstrated that high Se intake from the diet of broilers increased EPA, DHA and DPA concentration in thigh muscle [34]. They concluded that the dietary Se affects fatty acid concentration by increasing those enzymes activity involved in EPA and DHA synthesis from ALA or reducing EPA, DHA and DPA acids degradation rate by peroxidation processes. In another paper it has been shown that Se supplied in the maternal diet of chicks can protect DHA concentration in progeny’s tissues [35]. Moreover, another study showed the involvement of Se in the modulation of lipid metabolism [36]. Authors observed that rats with Se-adequate diet had higher omega-3 levels like DHA (but not EPA) and lower omega-6 levels in the liver phospholipids compared to rats with a Se-deficiency diet. These higher levels of DHA may be related to a direct effect of Se resulting in increased levels of selenoenzymes with antioxidative functions. Since they observed a differential effect of Se on different kinds of fatty acids such as EPA and DHA, they also suggested an indirect effect of Se on the absorption, storage and desaturation of fatty acids. Fatty acids such as LA and ALA are converted by elongation and desaturation to long chain PUFA. Δ-6 desaturase is the rate-limiting enzyme in the conversion process. It was reported that the activity of this enzyme is influenced by Se [37]. The increased concentration of LA and EPA in Se deficient rats might be caused by a decrease of their conversion to AA and to DHA respectively.

A similar Se role, in relation to omega-3 concentration in humans, has been observed in this study. The group eating KAMUT^®^ khorasan food received a 10-fold higher Se intake than the control group (Table 1). It is likely that this higher Se content in KAMUT^®^ khorasan products determined a reduction of DHA degradation rate by peroxidation and a higher activity of Δ-6 desaturase which determined a more efficient conversion of EPA to DHA. So the Se intake could be the main determinant of the higher serum DHA levels in KAMUT^®^ group. This “fish-like” effect in the absence of increased fish intake was described in a previous study where the authors reported the protective effect of moderate wine drinking comparable to that of fish in serum omega-3 levels in patients with CHD [38].

Data herein reported represent the first example of a direct effect of a variety of wheat, the KAMUT^®^ khorasan wheat, naturally high in Se, on DHA concentration. As reported [39], the modification of dietary patterns over the last 100–150 years has led to a change in fatty acid consumption, with a marked reduction in the consumption of omega-3. In particular Western societies tend to include very little fish in their diet and the authors suggest that an alternative for increasing the omega-3 fatty acid consumption would be to supplement with omega-3 daily foodstuffs. According to our study the KAMUT^®^ khorasan wheat could help in increasing the levels of omega-3 in the organism.

Previous papers reported whole grain protective effect against type-2 diabetes [40]. The basis of this protection is suggested to be cereal fiber, digested by the organism more slowly with respect to refined wheat. Furthermore, they observed that fasting insulin, a risk factor for type-2 diabetes mellitus, decreased with greater fiber intake, independent of energy and carbohydrate intake. For Diabetes Mellitus prevention, American Diabetes Association (ADA) recommends consumption of whole grains [41]. Moreover, epidemiological studies demonstrated that higher serum insulin levels are associated with an increased risk of colon, breast and other cancers [42]. The reduction of these insulin levels by whole grains may be an indirect way through which risk cancer reduction occurs [43].

In the present work, a whole grain diet based was utilized in both groups and the two types of whole grain were characterized by the same fiber content, but statistical significant decreased concentration of fasting insulin was observed only in KAMUT^®^ group. Moreover, a statistical significant decrease of Fat Mass, was obtained in KAMUT^®^ group, that plays a key role in diabetes development. As no drastic modifications on participants diet before recruitment were done, as confirmed by the fact that body weight of participants did not change, it could be supposed that the decrease of Fat Mass is related to the substitution of the type of cereal. It could be speculated that because the type of cereal fiber is highly important, the qualitatively different fiber present in ancient wheat like KAMUT^®^ khorasan wheat, as highlighted in a previous study [44], compared to a modern wheat, plays a key role in type-2 diabetes prevention. In fact, to reduce variability and to provide a similar fiber amount to each group, products from both kinds of wheat were prepared with the same technological process and had the same fiber content. Thus, we can hypothesize a possible role on the observed fat mass changes of the different kind of wheat fiber used and not of the different level of fiber intake between the two diets.

These findings support the hypothesis that type-2 diabetes risks factors such as low levels of plasma insulin are influenced by the type of wheat consumed (ancient or modern wheat), as shown in a previous study [16]. Moreover, it is likely that the improvements in insulin levels, observed only in KAMUT^®^ group, were mediated by the reduction in body fat mass in agreement with many previous studies [45].

The health benefits shown in the present work by a KAMUT^®^ khorasan wheat-based diet are further supported by the improvement of gut microbiota and metabolome in these volunteers as described previously [19]; in fact, the fecal and urine samples taken from each volunteer before and after the diet intervention showed that whole KAMUT^®^ khorasan-based diet was mainly characterized by the release of short-chain fatty acids (SCFA) and phenol compounds, as well as by a slight increase in health-promoting mutualists of the gut microbiota in comparison to whole durum wheat adopted as a control diet.

The present pilot study was subjected to limitations. The size of the patient population was small and larger studies are needed to verify results.

In conclusion, our study further confirms in vivo previous in vitro research on KAMUT^®^ khorasan wheat providing evidences regarding its protection against the development of type-2 diabetes in healthy volunteers, not evident in modern wheat varieties. However, further studies are necessary to confirm such hypothesis.
